# The first reported use of autologous blood pleurodesis for treatment of prolonged air leak in COVID‐19‐related spontaneous pneumomediastinum and pneumothorax: A case report

**DOI:** 10.1002/rcr2.840

**Published:** 2021-09-05

**Authors:** Muhammad Redzwan S. Rashid Ali

**Affiliations:** ^1^ KPJ Johor Specialist Hospital Johor Bahru Malaysia

**Keywords:** autologous blood pleurodesis, COVID‐19, prolonged air leak, spontaneous pneumomediastinum, spontaneous pneumothorax

## Abstract

Spontaneous pneumomediastinum (SPM) and pneumothorax (PTX) have been described as rare complications of COVID‐19 pneumonia. We present a case of COVID‐19 pneumonia which was complicated by SPM on Day 13 of admission with progression to spontaneous PTX 2 days later which necessitated intercostal chest drainage. It was complicated by prolonged air leak (PAL) for the next 9 days despite being on continued low‐dose suction and another additional larger bore intercostal drain inserted. Surgical pleurodesis was not an option in view of anaesthesia and operative risk expected in COVID‐19. In view of this, autologous blood pleurodesis (ABP) to address the alveolar pleural leak was opted. ABP has been previously used for PAL in cases of non‐COVID‐19‐related intractable spontaneous PTX. The air leak ceased with subsequent lung re‐expansion, with good clinical and radiological improvement. He was discharged well after resolution of PTX which required intercostal drain for a total of 15 days.

## Graphical abstract

This case describes the first reported use of autologous blood pleurodesis to treat prolonged air leak in a case of COVID‐19 pneumonia which was complicated by spontaneous pneumomediastinum and pneumothorax.

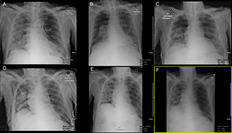

## INTRODUCTION

Spontaneous pneumomediastinum (SPM) and pneumothorax (PTX) can rarely complicate COVID‐19 pneumonia. It is self‐limiting in patients not requiring ventilatory support but in rare instances may require complex pleural intervention. It should be considered in the differential diagnosis of worsening disease in patients with COVID‐19.

This case describes the first reported use of autologous blood pleurodesis (ABP) to treat prolonged air leak (PAL) in a case of COVID‐19 pneumonia which was complicated by SPM and PTX.

## CASE REPORT

An 80‐year‐old gentleman with well‐controlled diabetes mellitus presented with fever and dry cough for 1 day. Clinical examination was unremarkable with blood pressure of 132/61 mmHg, pulse rate of 80 bpm, oximetry of 99% in room air and respiratory rate of 18 bpm.

A polymerase chain reaction nasal swab for COVID‐19 was positive and chest radiograph (CXR) revealed right lower lobe infiltrates consistent with COVID‐19 pneumonia. Treatment was started with oral favipiravir and empirical broad‐spectrum antibiotics. Intravenous dexamethasone and parenteral anticoagulation were added as he required supplemental oxygen (3 L/min) and remained fairly stable for the next 12 days of admission.

**FIGURE 1:**
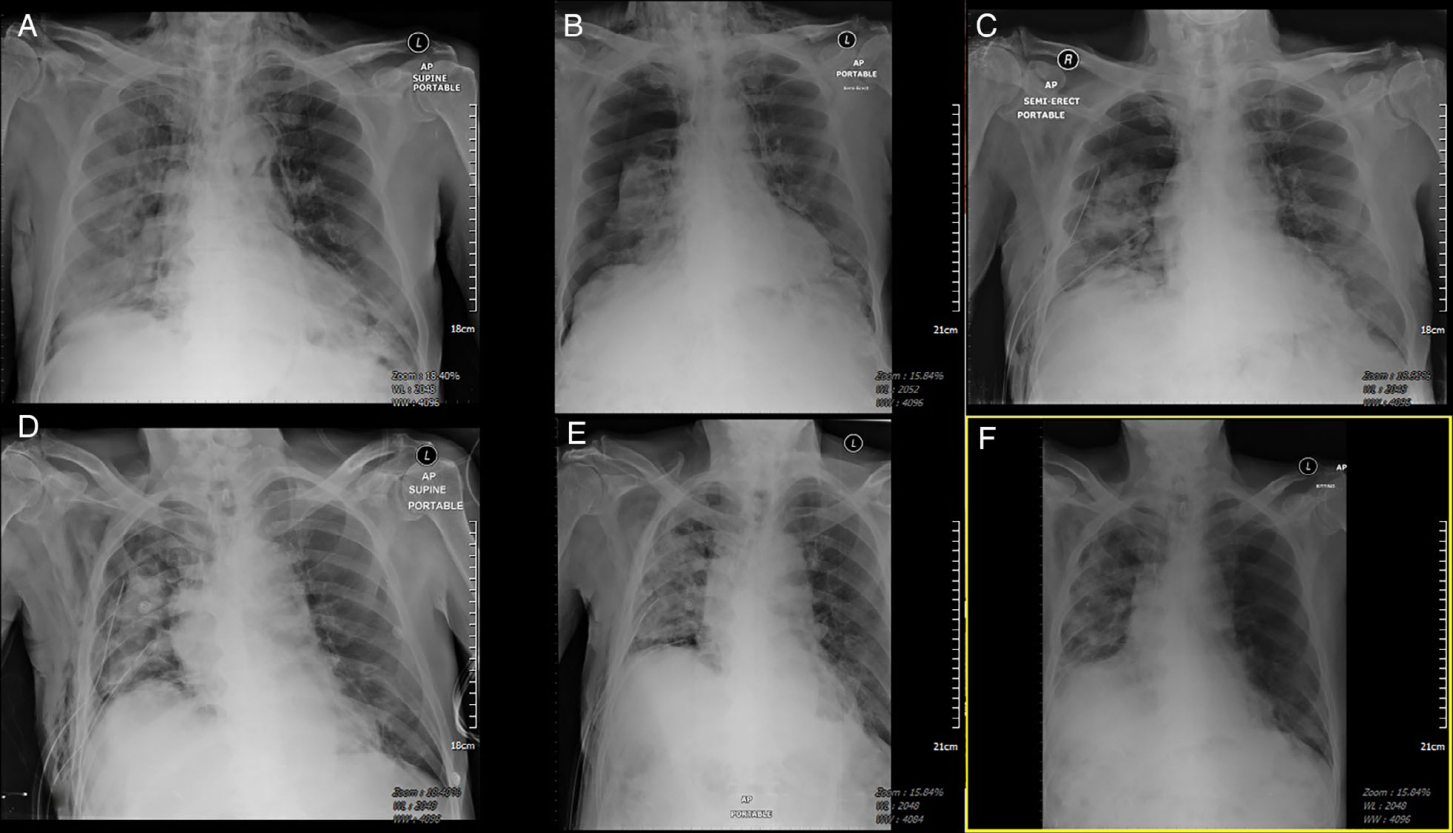
Serial chest radiograph. (A) Day 13 of illness with rim of air surrounding cardiac border (pneumopericardium) and air seen in subcutaneous tissue (subcutaneous emphysema [SCE]). (B) Day 15 of illness with large right pneumothorax (PTX). (C) Day 20 of illness with persistent air leak (5 days) and poor lung expansion. (D) Day 24 of illness with persistent air leak (9 days) and poor lung expansion with worsening SCE. (E) Day 26 of illness with cessation of prolonged air leak and lung fully re‐expanded after two doses of autologous blood pleurodesis. (F) Day 29 of illness with resolution of PTX and no recurrence after 24 h of removal of intercostal chest drain

**FIGURE 2:**
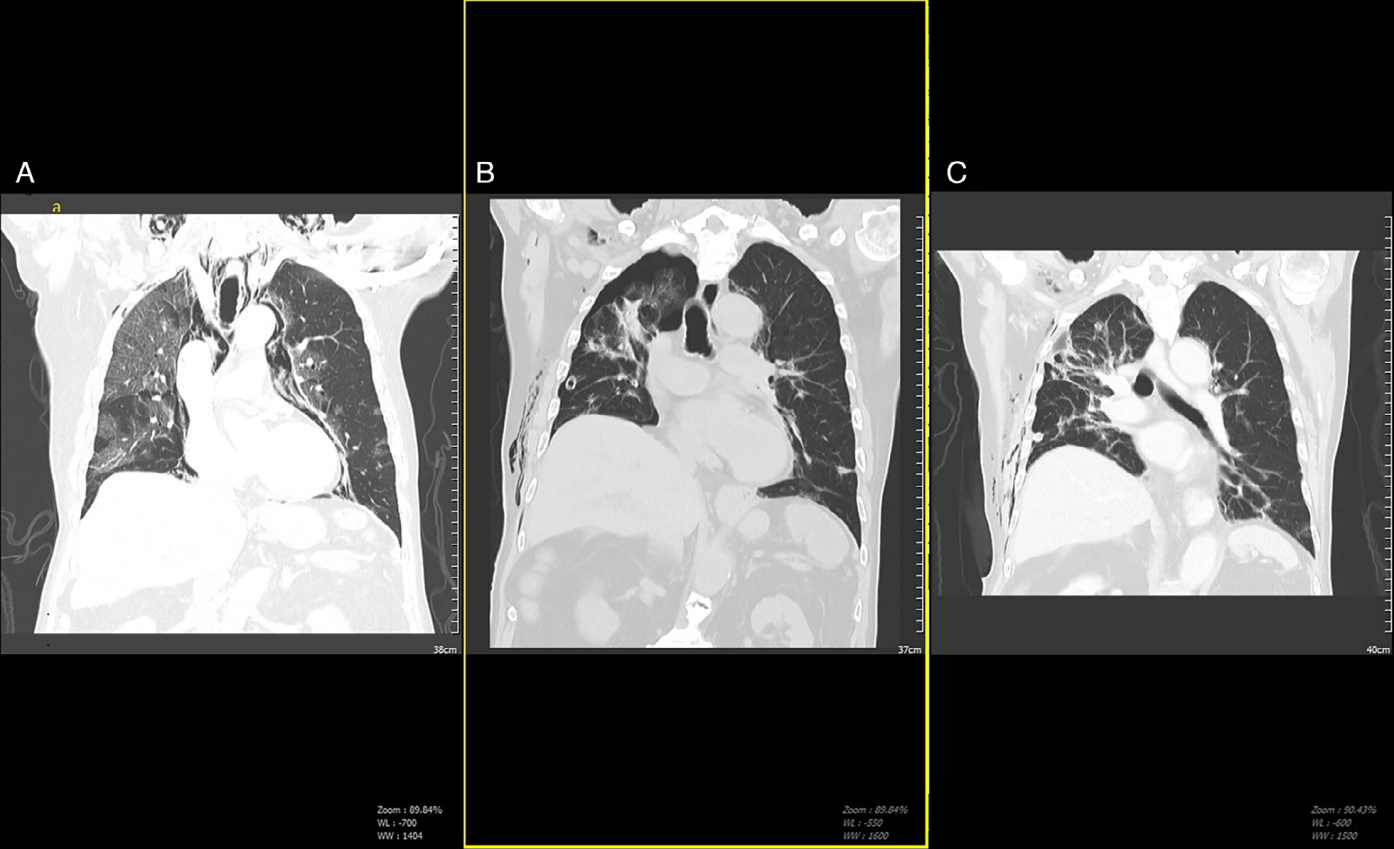
Serial computerized tomography thorax. (A) Day 13 of illness with pneumomediastinum pneumopericardium and subcutaneous emphysema, with diffuse ground‐glass opacity more prominent on the right lung parenchyma. (B) Day 24 of illness with persistent air leak and right pneumothorax (PTX) seen. (C) Day 28 of illness with resolution of PTX post autologous blood pleurodesis prior to removal of intercostal chest drain

On Day 13 of admission, he had some mild tightness of neck and clinical examination revealed palpable crepitus over the right chest wall and neck. CXR revealed mild pneumopericardium and subcutaneous emphysema (SCE) (Figure 1A). Haemodynamically, he was stable but oxygen requirement increased to face mask at 5 L/min. A computerized tomography (CT) thorax (Figure 2A) revealed extensive mild to moderate pneumopericardium, pneumomediastinum (SPM), SCE and generalized peripheral bilateral subpleural ground‐glass opacity consistent with COVID‐19 pneumonia. He was treated conservatively and was observed closely for any deterioration.

On Day 15 of admission, he developed sudden worsening breathlessness and repeat CXR (Figure 1B) revealed large right PTX. An urgent intercostal chest drain (ICD; size 24 Fr) was inserted and connected to underwater seal bottle with prominent air leak (Cerfolio Grade 4) noted. Low‐dose suction (5 cm water) was applied as CXR (Figure 1C,D) showed partial lung re‐expansion. The next 5 days showed continuous air leak with partial lung re‐expansion and a larger ICD (28 Fr) was inserted directed apically. Low‐dose suction was continued as the air leak was persistent, albeit with some improvement (Cerfolio Grade 2). The SCE and pneumopericardium resolved with residual upper PTX due to PAL lasting 9 days despite on optimal placing of two ICDs seen on subsequent interval of CT thorax (Figure 2B,C). This was treated conservatively as surgical intervention was deemed high risk at that time. In view of the anticipated prolonged PAL, he was planned for ABP in tandem with subsequent one‐way pneumostat chest drain valve (Heimlich, Atrium) application to the ICD to allow early ambulation for the patient.

ABP was done on Day 9 of PAL (two doses of 100 ml each on two consecutive days) and the air leak stopped 24 h after the second dose. He was well and without any oxygen supplementation after the cessation of air leak. Then, the smaller ICD was removed after full lung expansion a day later. The larger I then connected to the Heimlich valve for the next 3 days to allow for mobilization and to further encourage ambulation for the bedbound patient. The larger ICD was kept also to detect any potential recurrence of air leak and to avoid the need of repeated insertion if the PTX subsequently recurs; as per patient wishes.

Serial CXR (Figure 1E,F) showed full lung expansion with no recurrence of PTX and the larger ICD was then removed. The patient was discharged well and remains well with no recurrence on follow‐up.

## DISCUSSION

COVID‐19 infection (SARS‐CoV‐2) may present with respiratory complications such severe pneumonia and acute respiratory distress syndrome. Rare complications such as SPM and PTX have been recognized with diverse variable presentation.^1^, ^2^

Symptoms are usually non‐specific but can be missed easily such as chest pain, chest tightness, worsening shortness of breath, cough, dysphagia and crepitus over chest. Rupture of alveoli with subsequent air leak due to possible intense coughing is postulated to be the cause as described by Macklin phenomenon.^3^ Interstitial air from the rupture can potentially dissect into the mediastinum causing SPM, pneumopericardium and PTX, and also escape via subcutaneous tissue causing SCE. Majority of cases present within 2–3 weeks of diagnosis and, interestingly, patients do not have any pre‐existing lung disease nor require any positive pressure ventilation.^1^, ^3^ It is usually benign and self‐limiting without the need of invasive intervention in most cases. Treatment is usually supportive such as analgesic, oxygen supplement, bed rest, chest physiotherapy and treatment of the underlying COVID‐19 pneumonia.^3^

However, if it progresses to PTX, then the treatment principles should generally follow as for secondary spontaneous pneumothorax (SSP). In our patient, he developed PTX 2 days after the diagnosis of SPM, possibly due to COVID‐19‐related alveolar damage, microcyst and bullae formation, making it more prone to rupture.^3^ This may have contributed to the poor lung re‐expansion and prolongation of the alveolar–pleural leak despite on optimal adequate ICD with low‐dose suction therapy. Majority of COVID‐19‐related PTX have spontaneous resolution with conservative management; however, this was not the case in this patient as he had PAL.^3^

In view of high risk of surgical repair and anticipated PAL, ABP was attempted as the non‐surgical method of choice in this case. ABP has long been utilized in cases with PAL who are not fit for surgical repair in SSP.^4^, ^5^ ABP employs the sterile use of the patient's own blood that is injected intrapleurally (1–2 ml/kg each dose) to promote the cessation of alveolar pleural leak.^4^, ^5^ It has several advantages such as low rate of complications, inexpensive, easily done with the patient's own blood, can be utilized even if the lung is not expanded with a large air leak and causes less pleural adhesions. It is also the only non‐surgical technique which has been associated with rapid resolution of persistent air leak.^4^

This case describes a rare but an important pleural complication of COVID‐19 pneumonia. It also describes the first use of ABP as an option to treat PAL with good clinical outcome.

## CONFLICT OF INTEREST

None declared.

## ETHICS STATEMENT

Appropriate written informed consent was obtained for publication of this case report and accompanying images.
